# Socioeconomic and geographic variation in adjuvant chemotherapy among elderly patients with stage III colon cancer in Norway – a national register-based cohort study

**DOI:** 10.1007/s43999-024-00057-7

**Published:** 2024-12-17

**Authors:** Elin Marthinussen Gustavsen, Stig Norderval, Liv Marit Dørum, Aina Balto, Ragnhild Heimdal, Barthold Vonen, Eva Stensland, Ellinor Haukland, Beate Hauglann

**Affiliations:** 1 Department of Community Medicine, The Arctic University of Norway (UiT), Tromsø, Norway; 2 Centre for Clinical Documentation and Evaluation (SKDE), Northern Norway Regional Health Authority, Tromsø, Norway; 3 Department of Gastrointestinal Surgery, University Hospital of Northern Norway, Tromsø, Norway; 4 Department of Clinical Medicine, Faculty of Health Scienses, The Arctic University of Norway (UiT), Tromsø, Norway; 5 Cancer Registry of Norway, Norwegian Institute of Public Health, Oslo, Norway; 6 Geriatric Department, Akershus University Hospital, Lørenskog, Norway; 7 Northern Norway Regional Health Authority, Bodø, Norway; 8 Department of Oncology and Palliative Medicine, Nordland Hospital, Bodø, Norway; 9 SHARE – Center for Resilience in Healthcare, Faculty of Health Sciences, Department of Quality and Health Technology, University of Stavanger, Stavanger, Norway

**Keywords:** Socioeconomic variation, Geographic variation, Colon cancer, Adjuvant chemotherapy, Elderly, Health care utilisation

## Abstract

**Background:**

About half of the patients diagnosed with colon cancer are 70 years or older. Standard treatment for stage III colon cancer is major surgical resection followed by adjuvant chemotherapy (ACT). Norwegian guidelines recommend initiation of ACT within 6 weeks after resection.

**Objective:**

This study investigated socioeconomic and geographic variation in the recommended provision of ACT to elderly patients with stage III colon cancer in Norway.

**Methods:**

This population-based retrospective cohort study included patients aged 70 years or older diagnosed with stage III colon cancer between 2011 and 2021 who underwent major surgical resection. Individual data were obtained from national registries. Multilevel logistic regression analysis was used to model variation in provision of ACT.

**Results:**

Of 4 501 included patients, 603 (13%) and 1 182 (26%) received ACT within 6 and 8 weeks after resection, respectively. The provision of ACT decreased with increasing age and frailty. Odds of ACT within 6 weeks decreased for patients with low socioeconomic status (SES) compared to high SES (odds ratio (OR) 0.67 (95% confidence interval (CI) 0.50–0.91)), and decreased for patients living alone compared to those living with a cohabitant (OR 0.72 (95% CI 0.58–0.91)). Geographic variation was found between hospital referral areas (OR 0.41–2.58).

**Conclusions:**

Our study found that ACT provision to elderly stage III colon cancer patients is associated with SES and geography, indicating variation in guidelines adherence. Further research is needed to explore the impact of ACT timing among elderly patients with stage III colon cancer in Norway.

**Supplementary Information:**

The online version contains supplementary material available at 10.1007/s43999-024-00057-7.

## Introduction

Colon cancer is one of the most commonly diagnosed cancer types worldwide. The highest rates are seen in developed regions like Europe, Australia and Northern America [1]. In Norway, colon cancer is the fourth most commonly diagnosed cancer [2]. Of 3 385 patients diagnosed with colon cancer in 2022 [3], 64% were 70 years or older at diagnosis [4]. As the risk of colon cancer increases with age [5], this proportion is estimated to increase to 72% in 2040 due to the ageing population [6].

Standard treatment for stage III colon cancer is major surgical resection followed by adjuvant chemotherapy (ACT), a well-established treatment since the National Institutes of Health consensus in 1990 [7]. The consensus was based on clinical trials demonstrating improved survival associated with ACT [8, 9]. Confirmatory randomised studies were carried out in Norway, Sweden and Denmark in the 1990’s with similar findings [10, 11]. Elderly patients are often underrepresented in clinical trials [12]. Still, a pooled analysis of seven clinical trials and several cohort studies has indicated survival benefit from ACT for older patients as well [13–18].

The optimal time for initiating ACT has not been established by clinical trials. Norwegian guidelines recommend ACT to stage III colon cancer patients younger than 75 years of age within 4–6 weeks after resection [19]. Nevertheless, the guidelines recognise several studies indicating that ACT are most beneficial when initiated within 8 weeks after resection. For patients ≥ 75 years, ACT should be based on the patient’s individual health status, i.e. functional status, comorbidity and general condition according to the national guidelines.

Despite a well-established standard treatment, variation in the provision of ACT has been observed in several countries [14, 20–23]. Age and comorbidity have consistently been demonstrated as strong predictors for ACT [14, 16, 17, 22, 24, 25]. Studies have also shown socioeconomic disparities in the provision of ACT where higher socioeconomic status (SES) is associated with increased provision of ACT [20, 22–24]. Geographic variation in the provision of ACT has received less attention, although between-hospital variation [20, 23] as well as variation between rural and urban regions [21, 26] have been observed.

Four regional health authorities are responsible for the provision of all specialised health care in Norway. They own hospital trusts of varying size, which provide specialist services to the population living within their hospital referral area (HRA). The health care is tax-funded and in principle free of charge for all Norwegians [27]. All hospital trusts provide major resection and ACT to colon cancer patients. The decision on whether to provide ACT to stage III colon cancer patients is made by an oncologist and/or a surgeon. The Norwegian Colorectal Cancer Registry reported that 17% of elderly patients (≥ 75 years) who underwent resection for stage III colon cancer in 2019–2021, were treated with ACT, varying from 4 to 30% between the hospital trusts [3].

Cancer management in the oldest patients requires individual assessment due to their diverse health statuses. This may increase variation in clinical practice. There is limited information on the socioeconomic and geographic variations in the administration of ACT within 6 to 8 weeks for elderly patients with stage III colon cancer. Consequently, this study aims to assess adherence to national guidelines for ACT provision among elderly patients with stage III colon cancer in Norway and to explore potential socioeconomic or geographic variations.

## Methods

### Study design and data sources

**Fig. 1:**
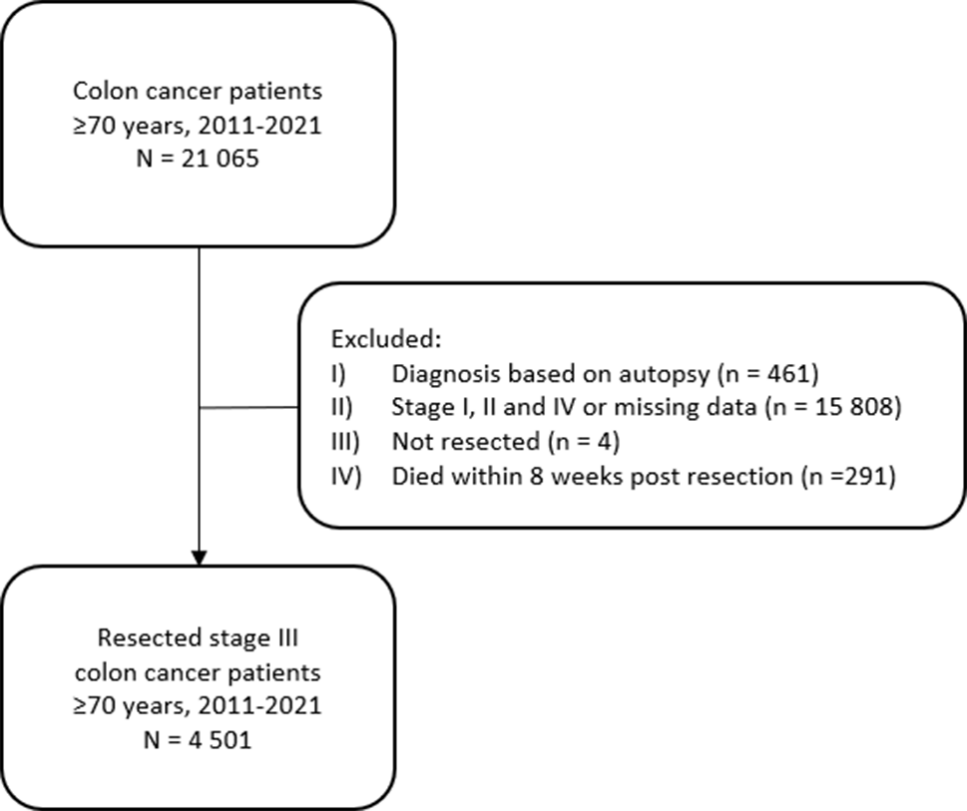
Flowchart showing inclusion of patients in the study

This national register-based cohort study included all Norwegians aged 70 years or older, diagnosed with stage III colon cancer in the period 1 January 2011 to 31 December 2021 and who had undergone major resection of the cancer, and were eligible for ACT within 8 weeks (Fig. 1).

Patients with stage I, II or IV colon cancer, or missing data on stage, were omitted from the study (Fig. 1). Further, individuals were excluded from analysis if their cancer diagnosis was based solely on death certificate or autopsy, if they had no major surgical resection or if they died within 8 weeks after the resection.

Individual-level data were obtained from national health and administrative registries with complete coverage and linked by encrypted serial numbers derived from the personal identity number held by all Norwegian citizens.

The Cancer Registry of Norway (CRN) is a national registry to which reporting of all cancer cases is mandatory. The CRN has data of high quality and a high degree of completeness [28]. For the present study, the CRN extracted data on all patients aged 70 years or older diagnosed with cancer in the period 1 January 2011 to 31 December 2021, and provided data on age, sex, cancer diagnosis, date of diagnosis and basis for diagnosis. Patients with colon cancer were identified via the International Classification of Diseases 10th Revision (ICD-10) code C18. Data on pathological stage and resection were obtained from The Norwegian Colorectal Cancer Registry, a clinical registry within the CRN, which has collected data on colon cancer since 2007.

The Norwegian Patient Registry (NPR) holds data on visits to specialist health care services, like public hospitals and contracted private institutions and specialists. Use of primary health care services, such as general practitioners and out-of-hours services, are registered in the Norwegian Control and Payment of Health Reimbursements Database (KUHR). Data registered on the study population from 1 January 2010 through December 2022, including diagnoses, procedures, treating hospitals, time and type of visit were received from NPR, and date of contact and International Classification of Primary Care (ICPC) codes from KUHR.

Statistics Norway (SSB), the national statistics office, provided information on yearly demographic and socioeconomic data with residential information, education, income, and type of household.

### Definitions

The primary outcome of this study was receipt of ACT initiated within the first 6 weeks after resection. The secondary outcome was receipt of ACT within the first 8 weeks after resection. ACT was identified by the diagnosis code Z51.1* or the procedural codes WBOC05, WBOC08 and WBOC20.

Stage III was defined according to the pathological tumour-node-metastasis (TNM) classification: any pT, pN1-2 and pM0. This includes all cases where the tumour has spread to regional lymph nodes (N1-2), but not to distant metastatic sites (M0).

Age at cancer diagnosis was categorised into three groups; 70–74, 75–79, and ≥ 80 years. Frailty was measured by a frailty index (FI) based on primary care data [29]. ICPC codes within 12 months prior to the cancer diagnosis were used to calculate each individual’s FI. These codes include “health deficits” such as diseases, and psychosocial or functional impairments which would represent the elderly patient’s diverse health conditions better than comorbidity. The FI was categorised into three scores; low (0–1), intermediate (2–3) and high score (≥ 4). Urgency of resection was categories as acute or not.

The patient’s SES was measured as a composite score based on a weighted combination of education and personal income [30]. The patient’s highest obtained education was categorised according to four levels: primary education (< 10 years), upper secondary/vocational, undergraduate degree and postgraduate degree. Yearly after-tax personal income in the year prior to the year of diagnosis, was consumer price index (CPI) adjusted and divided into sex-specific quartiles. Empirically derived weights for individuals aged ≥ 66 years were then applied from Lindberg et al. [30] to combine the education and income level into a composite score categorised into low, intermediate and high SES.

Type of household was categorised as living alone, living with a cohabitant and not living in private household (e.g. nursing facilities).

Municipal or city district residency in the year prior to the colon cancer diagnosis defined the patient’s HRA affiliation.

### Statistical analyses

Descriptive statistics were used to describe patient characteristics. Multilevel logistic regression analyses were conducted to estimate odds ratios (ORs) for comparisons across the socioeconomic groups and HRAs. Age-stratified analysis was conducted for the three age groups. Directed acyclic graphs (DAGs) were used in separate analyses to avoid Table 2 fallacy [31].

In the multilevel analysis, an empty model (null model) with cluster-specific random effects only was initially applied to model variation between HRAs. The intraclass correlation coefficient (ICC) was calculated to quantify the proportion of the total variance in the provision of ACT that was attributable to the HRA level [32]. To investigate whether the geographic variation could be attributed to other patient factors and socioeconomic variation, two further models were examined. Model 1 incorporated patient characteristics, including sex, age and frailty. Model 2 (full model) extended model 1 by adding SES, type of household and year of diagnosis. Other variables, like children and their education and place of residence, and travel time to nearest treating facility, were explored, but excluded in the full model since they were not significant and did not improve the model. All models were examined for multicollinearity by inspecting correlation and variance inflation factors.

All statistical analyses were conducted using SAS V.9.4 (SAS Institute).

In the following section, descriptive analysis is presented followed by analyses stratified by ACT timing. The primary focus is on analyses of ACT initiation within 6 weeks after resection, in accordance with national guidelines.

## Results

A total of 4 501 patients aged 70 years or older, diagnosed with stage III colon cancer in the period 2011–2021 and who had undergone a major resection, were included in this study (Table 1). Age at diagnosis ranged from 70 to 99 years, with a mean age of 79.0 years. Among the patients, 1 644 (37%) had low frailty score, 546 (12%) had high SES and 2 669 (59%) were living with a cohabitant.

**Table 1:**
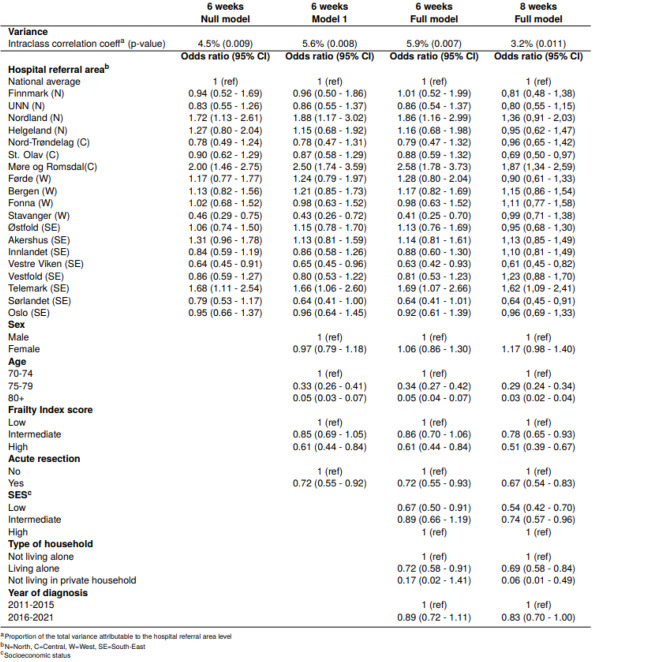
Multilevel logistic regression of three models with odds ratio and 95% confidence interval for adjuvant chemotherapy within 6 weeks after resection and of full model for adjuvant chemotherapy within 8 weeks after resection. Intraclass correlation coefficient of hospital referral area with p-value. Model 1: adjusted for demographic and clinical characteristics. Full model: model 1 + adjusted for socioeconomic factors

**Fig. 2:**
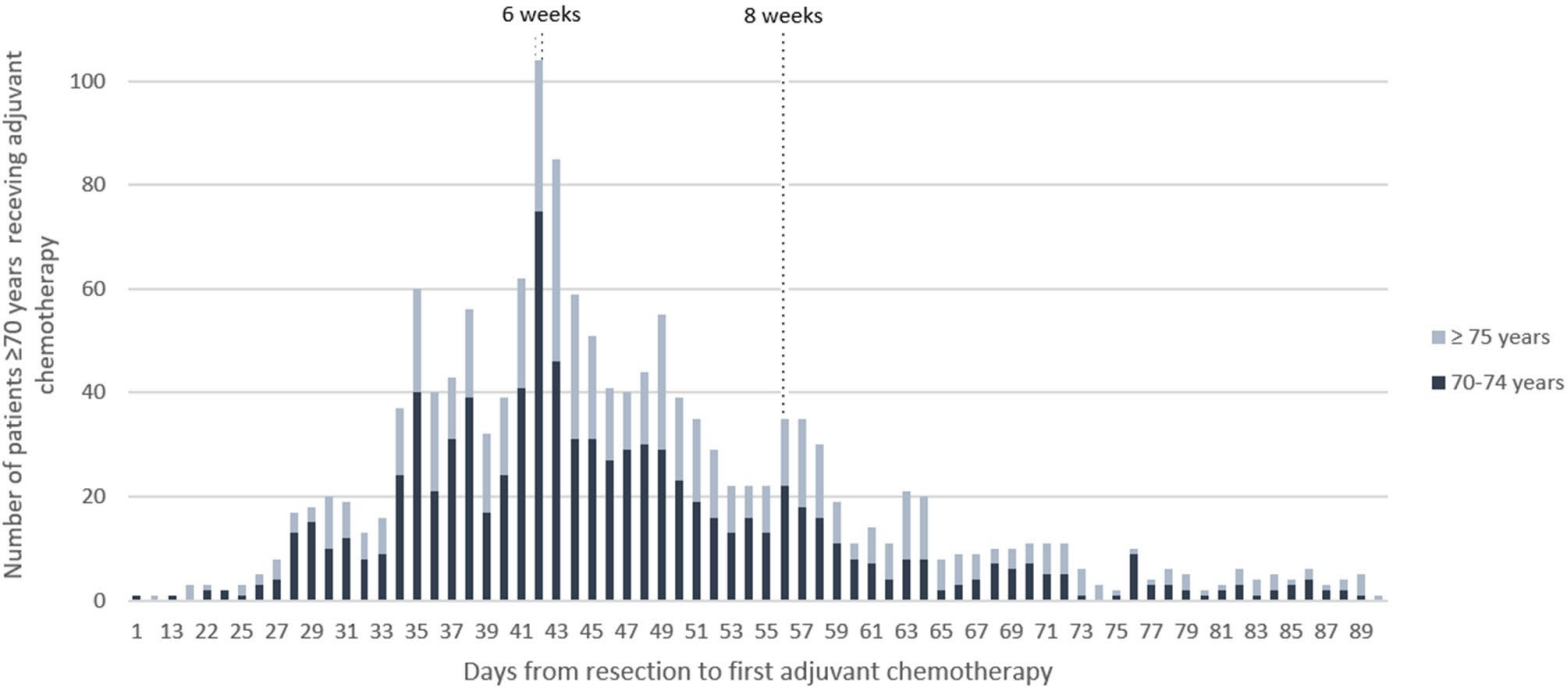
Number of elderly patients, 70–74 years (dark blue) and ≥ 75 years (light blue), with stage III colon cancer by days from resection to first adjuvant chemotherapy

Figure 2 shows days from resection to the initiation of ACT within the first 90 days after resection.

### ACT within 6 weeks

In total, 603 (13%) were provided ACT within 6 weeks after resection (Table 2). The proportion decreased with advancing age from 31% in age group 70–74 to 2% in patients aged ≥ 80 years. It also decreased from 17 to 8% as frailty increased from low to high score. The proportion receiving ACT increased from 11 to 18% as SES increased from low to high. Patients living alone had a lower proportion receiving ACT compared to those living with a cohabitant (9% vs. 17%).

**Table 2:**
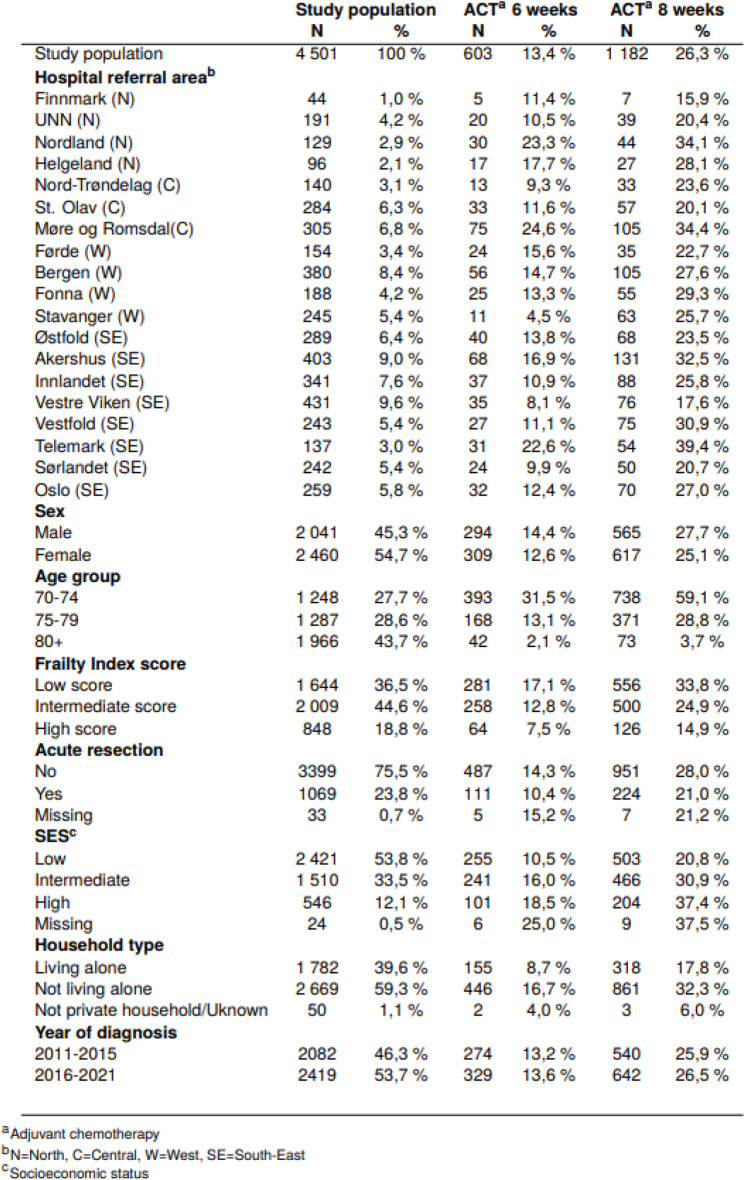
Characteristics of elderly patients (≥ 70 years) diagnosed with stage III colon cancer in Norway in 2011–2021 and who had undergone major resection, in total and by adjuvant chemotherapy provided to patients within 6 and 8 weeks after resection

#### Age and frailty

Both advancing age and high frailty were associated with decreasing odds of ACT within 6 weeks (Table 1). In the fully adjusted model, patients aged 75–79 years had 66% lower odds of ACT than patients aged 70–74 years (OR 0.34 (95% CI 0.27–0.42)). The odds decreased further for patients ≥ 80 years. Odds of ACT decreased by 39% for patients with high frailty compared with patients with low score (OR 0.61 (95% CI 0.44–0.84)).

#### Socioeconomic variation

Patients with low SES and patients living alone were less frequently treated with ACT within 6 weeks (Table 2). Patients with low SES had 33% lower odds of having ACT than those with high SES (OR 0.67 (95% CI 0.50–0.91)) (Table 1). Patients living alone had 28% lower odds of receiving ACT compared to those living with a cohabitant (OR 0.72 (95% CI 0.58–0.91)).

In the age-stratified analysis, the provision of ACT within 6 weeks was associated with SES and type of household among patients aged 75–79 years (Table 3). Patients aged 75–79 with low SES had 49% lower odds of ACT compared to those with high SES (OR 0.51 (95% CI 0.30–0.88)), while those living alone had 44% lower odds compared to those living with a cohabitant (OR 0.56 (95% CI 0.36–0.86)).

**Table 3:**
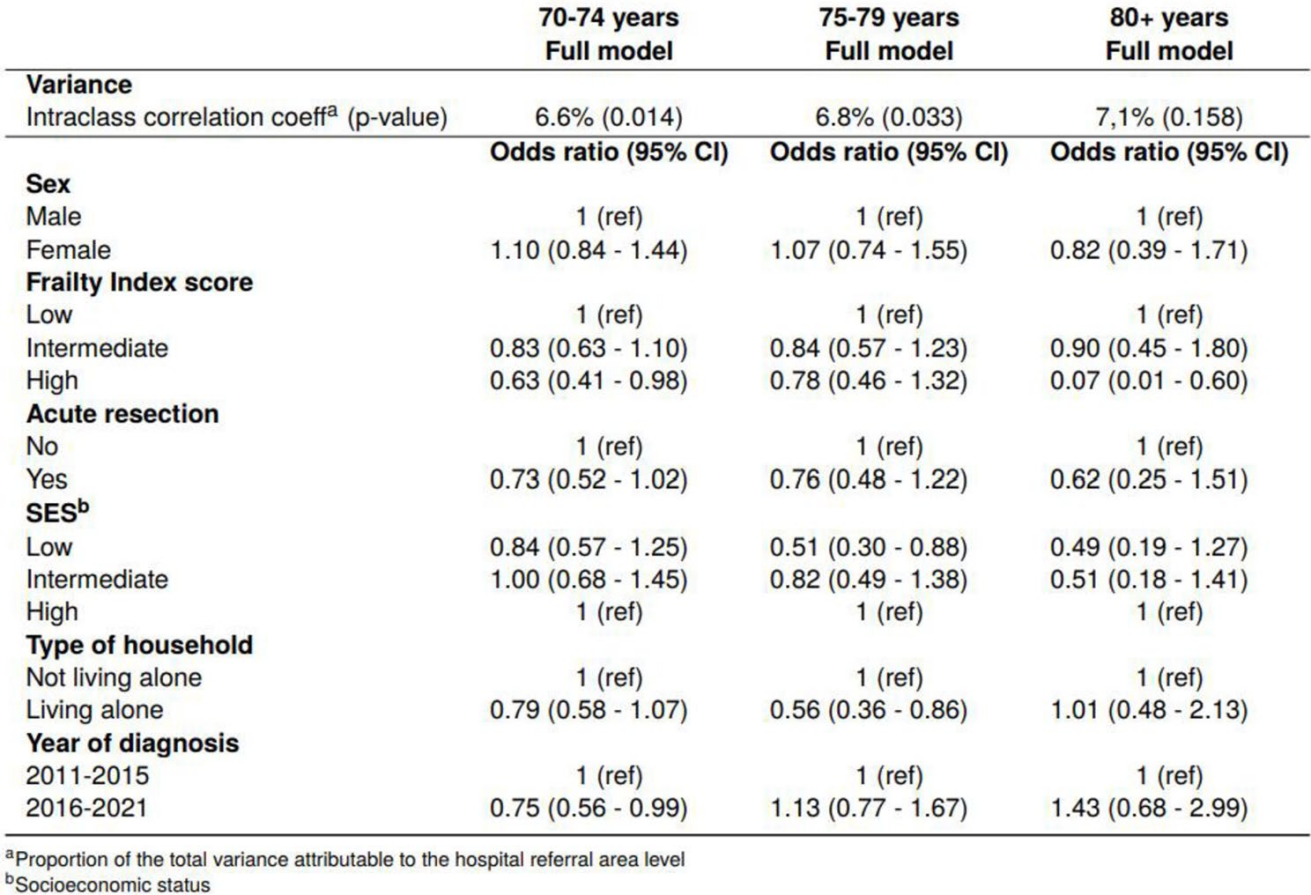
Multilevel logistic regression of the full model with odds ratio and 95% confidence interval for adjuvant chemotherapy within 6 weeks after resection by age groups. Intraclass correlation coefficient of hospital referral area with p-value

#### Geographic variation

**Fig. 3:**
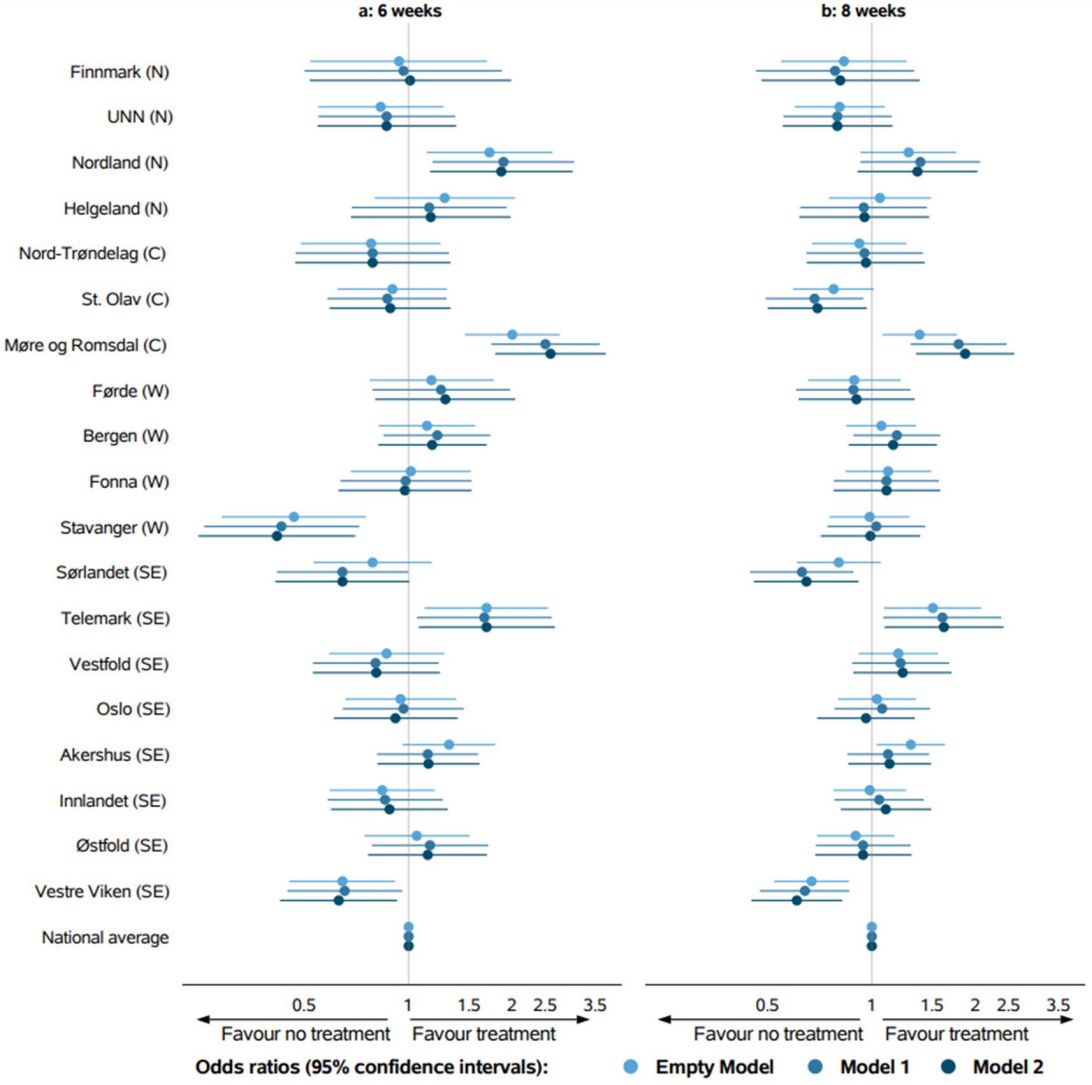
Odds ratios with 95% confidence intervals for adjuvant chemotherapy within a: 6 weeks and b: 8 weeks after resection for each hospital referral areas grouped by the four regional health authorities: North (N), Central (C), West (W) and South-East (SE). Model 1: adjusted for demographic and clinical characteristics, Model 2: Model 1 + adjusted for socioeconomic factors

The proportions treated with ACT within 6 weeks ranged from 4 to 25% between the HRAs. ORs and 95% confidence intervals (CIs) for the HRAs for all models are depicted in Fig. 3.

Geographic variation across the HRAs in the provision of ACT within 6 weeks were present in the null model (Fig. 3). Moreover, the ORs remained largely unchanged when introducing clinical and demographic factors in model 1 and socioeconomic factors in model 2. In the fully adjusted model, ORs ranged from 0.41 (95% CI 0.25–0.70) to 2.58 (95% CI 1.78–3.73) for provision of ACT compared to national average. Overall, patients in three HRAs had significant higher odds of ACT compared to the national average, whereas patients in two HRAs had significant lower odds. Overall, 5.9% of the geographic variation was attributable to the HRA level (Table 1).

**Fig. 4:**
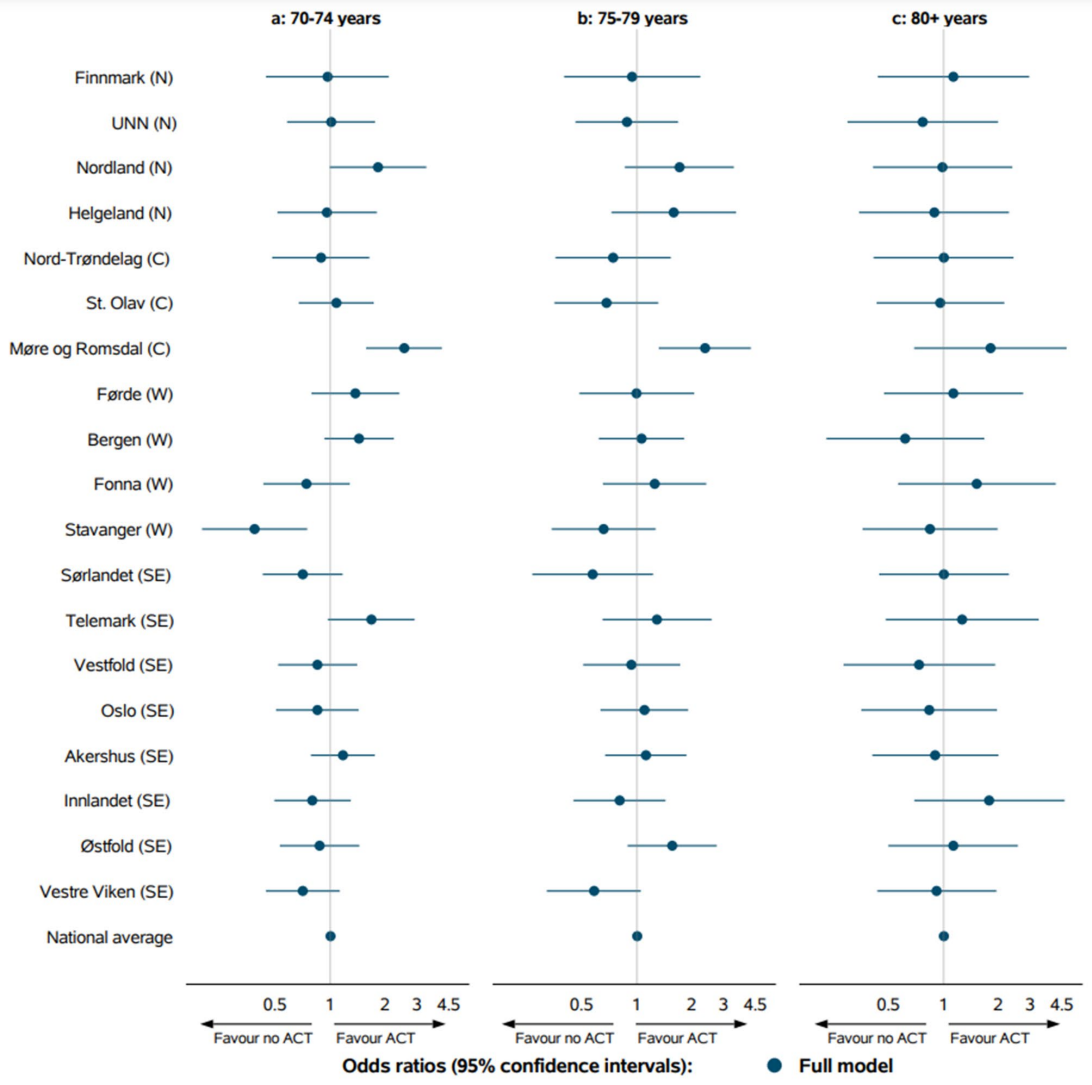
Odds ratios with 95% confidence intervals for the full model for adjuvant chemotherapy within 6 weeks after resection for each hospital referral areas grouped by the four regional health authorities: North (N), Central (C), West (W) and South-East (SE) by age: a 70–74 years, b 75–79 years and c 80 years or older

The proportions receiving ACT within 6 weeks across the HRAs ranged between 8 and 57%, 4–29% and 0–5% for patients aged 70–74, 75–79 and ≥ 80 years, respectively (Online Resource 1). In the fully adjusted model, significant variation between HRAs was found for patients aged 70–74 and 75–79 years (Fig. 4).

### ACT within 8 weeks

**Fig. 5:**
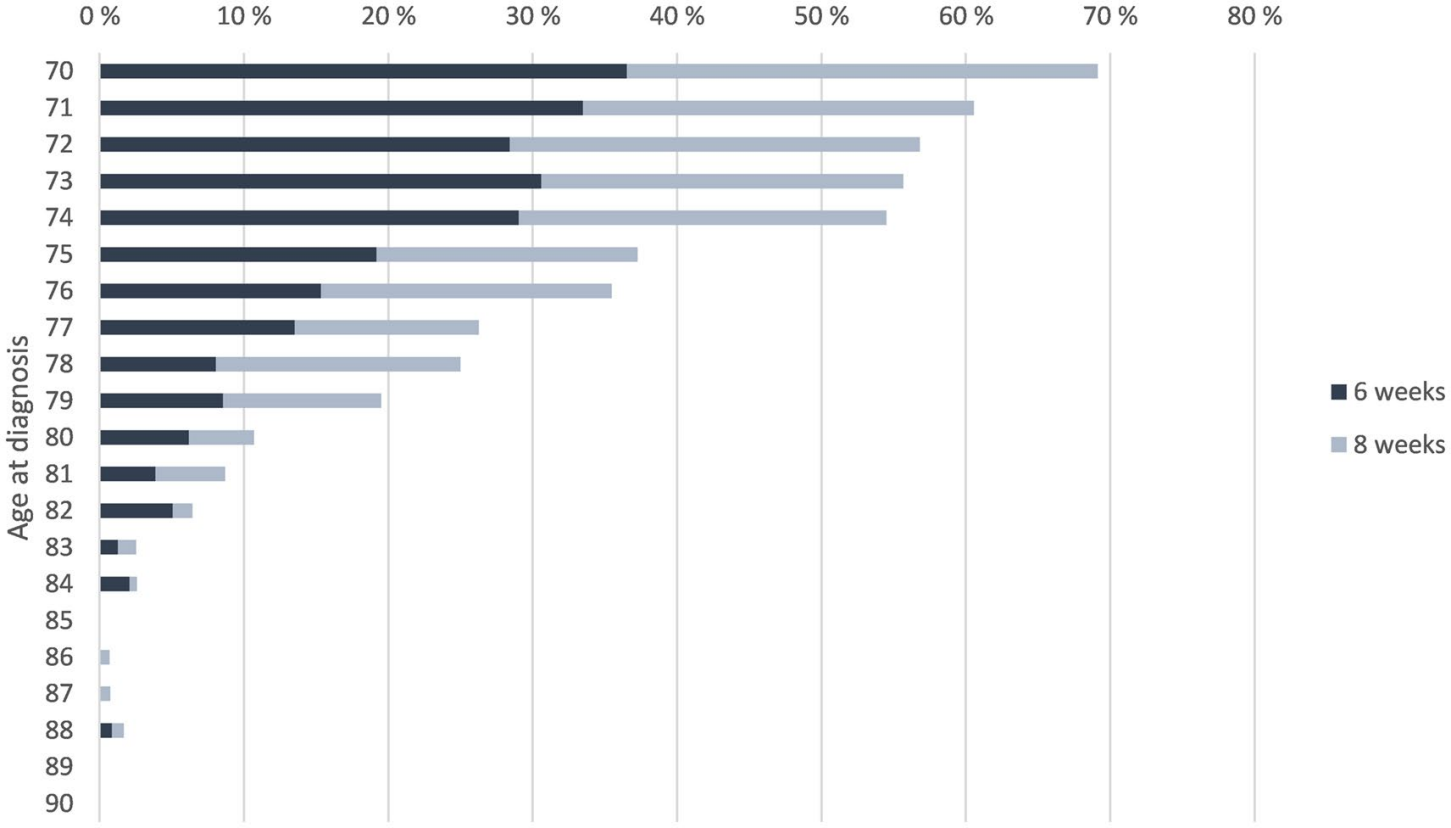
The proportion of stage III colon cancer patients receiving ACT within 6 and 8 weeks after resection by age of diagnosis

In total, 1 182 patients (26%) received ACT within 8 weeks after resection (Table 2). Almost a twofold increase in provision of ACT within 8 weeks was observed across nearly every age compared to within 6 weeks (Fig. 5).

#### Socioeconomic variation

The associations for ACT within 8 weeks were similar, but somewhat stronger, to those found for provision of ACT within 6 weeks (Table 1). Patients with low SES had 46% lower odds of having ACT than those with high SES (OR 0.54 (95% CI 0.42–0.70)). Patients living alone had 31% lower odds of receiving ACT compared to those living with a cohabitant (OR 0.69 (95% CI 0.58–0.84)).

#### Geographic variation

The proportions treated with ACT within 8 weeks ranged from 16 to 39% between the HRAs (Table 2). In the fully adjusted model, ORs ranged from 0.61 (95% CI 0.45–0.82) to 1.87 (95% CI 1.34–2.59) (Fig. 3). Overall, 3.2% of the geographic variation was attributable to the HRA level (Table 1).

## Discussion

This study shows both socioeconomic and geographic variation in the provision of ACT to elderly patients with stage III colon cancer in Norway. Patients with high SES and patients living with a cohabitant receive ACT more frequently than those with low SES and those living alone. To our knowledge, this is the first study exploring both socioeconomic and geographic variation in the provision of ACT within 6 and 8 weeks among elderly patients with stage III colon cancer by the use of individual data.

### Adherence to guidelines

The Norwegian guidelines recommend that ACT should be initialised within 4–6 weeks after major resection for patients younger than 75 years. Nevertheless, our study shows that more than two out of three patients aged 70–74 years do *not* receive ACT within 6 weeks. Similar studies, but with different age and time limits, found that about 40% of patients aged 70–79 years from Sweden and the Netherlands did not receive ACT [16]. A study from England found that about 30% of patients aged 70–74 years did not receive ACT within 4 months [16, 20].

Our study shows that an equal number of patients receive the first ACT in week 7 and 8 after resection as within the first 6 weeks. This finding indicates a clinical practice where the initiation of ACT is equally likely to occur in week 7 and 8 as within the first 6 weeks. Furthermore, one out of five patients receiving ACT start with adjuvant treatment later than 8 weeks after resection. Similar result was found in a Norwegian study were 14.5% of patients below the age of 75 received their first ACT later than 8 weeks after resection [33].

Several studies have found an elevated mortality in those who started ACT later than 8 weeks after resection [34–36]. A meta-analysis comparing delayed ACT to standard care, found that delaying initiation of ACT beyond 8 weeks after resection was associated with worse overall survival (risk ratio: 1.20) [37]. Although the national guidelines recommend initiation of ACT within 6 weeks, they also acknowledge multiple studies indicating that ACT is most beneficial when initiated within 8 weeks after resection. Further research is needed to understand why some elderly patients are provided ACT long after the recommended guidelines.

### Age and frailty

The present study shows that older age and high frailty are independently associated with decreasing odds of receiving ACT, consistent with findings from other studies [17, 20, 22–24, 38]. These results are in line with the guidelines stating that patients aged 75 years or above should be individually assessed based on their health status on whether to provide ACT or not. A decrease in odds of ACT due to both age and frailty was therefore expected.

### Socioeconomic variation

Our study finds a significant association between SES and provision of ACT. Patients with high SES are more likely to receive ACT compared to those with low SES. This is similar to findings of numerous studies discussing socioeconomic variation in the ACT management of stage III colon cancer patients [17, 20, 22–24]. In a systematic review and meta-analysis, Konradsen et al. [39] found that low SES, regardless of indicator, was associated with less initiation of ACT to stage III colon cancer patients.

The observed association between SES and provision of ACT may be influenced by health literacy. This can be defined as the “ability to find, understand, and use information and services to inform health-related decisions and actions for themselves and others” [40]. It has been found to follow a social gradient, where patients of lower SES are more likely to have limited health literacy [41, 42]. This could affect their capacity to comprehend provided information and make informed treatment decisions together with their physicians. Nevertheless, clinicians have the responsibility to ensure that their patients understand their treatment options. Employing shared decision-making tools may enhance patient knowledge and awareness of available treatment choices [43].

Moreover, studies have demonstrated a social gradient in doctor-patient communication, where physicians provide less information and engage in less participatory communication with patients of lower SES [44, 45]. This gradient in doctor-patient communication could be explained by the patient’s communicative style. Patients with lower SES are found to be less active when communicating with their physician [46]. They ask fewer questions and are less opinionated compared to those with higher SES. This communication disparity could potentially influence physicians’ decision on providing ACT, presuming lower independence and less compliance to a treatment regime among patients with lower SES [47].

Our study also shows that patients living alone are less likely to receive ACT compared to those living with a cohabitant. This is consistent with other studies indicating that marriage is associated with provision of ACT [21, 38]. Hu et al. [48] found that married patients ≥ 65 years had 79% higher odds of ACT compared to unmarried patients. As people age, cognition declines, and the ability to maintain functional independence might be harder to uphold [49]. The association between marriage or living with a cohabitant, and receipt of ACT suggests an important role of a partner in ACT management of elderly patients. Physicians might be worried about the patient’s ability to manage a chemotherapy regime and therefore be hesitant to provide chemotherapy to patients with cognitive impairment who live alone.

### Geographic variation

This study shows geographic variation in the provision of ACT across the HRAs. Other studies have documented between-hospital variation in the provision of ACT for stage III colon cancer in England [20] and the Netherlands [23], while other have found variation between urban and rural regions in the US [21] and Australia [26].

The intraclass correlation coefficient in our study is low, indicating that little of the total individual variation can be explained by factors at the HRA level. Instead, it is likely due to individual factors, such as the preferences of physicians or patients. Some physicians might differ in how they assess patients’ health statuses or in how restrictive they are in management of ACT to elderly patients. Keating et al. [50] found that physicians differed widely in recommendations of ACT to patients who are older and sicker. Additionally, a systematic review found that physicians’ recommendations were the most important factor for older adults’ acceptance of cancer treatment [51].

The geographic variation in the provision of ACT within 6 and 8 weeks differ somewhat. In certain areas, the odds of providing ACT within 8 weeks are higher than within 6 weeks, and vice versa. Other areas maintain consistently low or high odds regardless of when ACT is initiated. This may suggest differing practices in the interpretation of the guidelines.

### Strengths and limitations

A major strength of this study is the utilisation of individual-level data from national registries renowned for their high quality and completeness. This enabled the inclusion of crucial factors such as frailty and SES at the individual level in our analyses. Consequently, our study yields unique insights and produces results that are widely representative.

There are some limitations to this study. Despite the high completeness of the data, a small proportion of cancer staging was missing. This could introduce selection bias. However, we do not anticipate systematic variation in the missing data. Additionally, ACT was identified based on chemotherapy codes irrespective of regimen. Some cases might therefore include atypical practice or palliative treatment. However, given the large sample size and exclusion of stage IV patients, the number of patients eventually having received palliative chemotherapy within 6 or 8 weeks after resection is believed to be very low and not affecting the results. Another notable limitation is the absence of data on patient factors like cognitive function, nutritional status and patients’ own preferences, that may influence provision of ACT. Since our study does not account for patients’ preferences, we cannot conclusively claim that the variation are entirely unwarranted [52].

## Conclusion

Despite Norway’s universal and public healthcare system, set to ensure equal healthcare access irrespective of SES or place of residence, our study unveils variation in provision of ACT among elderly patients with stage III colon cancer, both with regards to SES and place of residence. Our findings indicate a variation in adherence to treatment guidelines. Further research is needed to investigate the impact of ACT timing on outcomes for elderly patients with stage III colon cancer in Norway.

## Electronic supplementary material

Below is the link to the electronic supplementary material.

Supplementary Material 1

## Data Availability

The data underlying this article cannot be shared publicly due to legal restrictions.
