# Supplementary material for: Socioeconomic and geographic variation in adjuvant chemotherapy among elderly patients with stage III colon cancer in Norway – a national register-based cohort study
Source: Res Health Serv Reg. 2024 Dec 17;3:21. doi: 10.1007/s43999-024-00057-7 (PMC11652436; doi:10.1007/s43999-024-00057-7)
Supplement: Supplementary file 1 — Supplementary Material 1 [file 43999_2024_57_MOESM1_ESM.pdf]

Online Resource 2: Proportions of patients provided adjuvant chemotherapy within 6 weeks (1) and 8 weeks (2) after resection for the hospital referral areas by three age groups: 70-74 years (a), 75-79 years (b) and ≥80 years (c).

	**1 Adjuvant chemotherapy within 6 weeks post resection**		
	**a 70-74 years**	**b 75-79 years**	**c 80years**
**Total**	31,5 %	13,1 %	2,1 %
**Hospital referral area**			
Finnmark (N)	27,3 %	8,3 %	4,8 %
UNN (N)	29,8 %	10,2 %	0,0 %
Nordland (N)	52,9 %	28,9 %	1,8 %
Helgeland (N)	33,3 %	24,0 %	0,0 %
Nord-Trøndelag (C)	27,3 %	7,1 %	1,5 %
St. Olav (C)	32,9 %	6,1 %	1,6 %
Møre og Romsdal(C)	56,6 %	28,0 %	4,8 %
Førde (W)	40,0 %	12,1 %	2,6 %
Bergen (W)	39,6 %	15,8 %	0,0 %
Fonna (W)	22,8 %	16,3 %	4,9 %
Stavanger (W)	8,3 %	6,3 %	0,9 %
Østfold (SE)	28,2 %	20,0 %	2,3 %
Akershus (SE)	34,0 %	13,8 %	1,5 %
Innlandet (SE)	24,7 %	8,6 %	4,6 %
Vestre Viken (SE)	23,8 %	6,5 %	1,5 %
Vestfold (SE)	26,4 %	11,4 %	0,0 %
Telemark (SE)	45,8 %	17,9 %	4,0 %
Sørlandet (SE)	21,0 %	3,5 %	4,8 %
Oslo (SE)	27,0 %	15,7 %	0,9 %

	**2 Adjuvant chemotherapy within 8 weeks post resection**		
	**a 70-74 years**	**b 75-79 years**	**c 80years**
**Total**	59,1 %	28,8 %	3,7 %
**Hospital referral area**			
Finnmark (N)	45,5 %	8,3 %	4,8 %
UNN (N)	55,3 %	20,3 %	1,2 %
Nordland (N)	64,7 %	50,0 %	5,3 %
Helgeland (N)	57,6 %	32,0 %	0,0 %
Nord-Trøndelag (C)	66,7 %	19,0 %	4,6 %
St. Olav (C)	54,4 %	12,2 %	3,3 %
Møre og Romsdal(C)	74,7 %	44,0 %	6,8 %
Førde (W)	62,2 %	15,2 %	2,6 %
Bergen (W)	65,3 %	36,6 %	1,1 %
Fonna (W)	56,1 %	36,7 %	6,1 %
Stavanger (W)	58,3 %	30,4 %	3,8 %
Østfold (SE)	52,1 %	30,6 %	3,8 %
Akershus (SE)	60,4 %	33,3 %	2,2 %
Innlandet (SE)	58,8 %	27,6 %	6,0 %
Vestre Viken (SE)	47,5 %	17,7 %	2,9 %
Vestfold (SE)	66,7 %	37,1 %	1,0 %
Telemark (SE)	68,8 %	46,2 %	6,0 %
Sørlandet (SE)	43,2 %	17,5 %	4,8 %
Oslo (SE)	63,5 %	27,0 %	5,6 %
